# Effect of linearly polarized microwaves on nanomorphology of calcium carbonate mineralization using peptides

**DOI:** 10.1038/s41598-023-37473-7

**Published:** 2023-07-25

**Authors:** Kenji Usui, Makoto Ozaki, Kan Hirao, Tsubasa Kosaka, Natsumi Endo, Shuhei Yoshida, Shin-ichiro Yokota, Yonejiro Arimoto, Ryuji Osawa, Nobuhiro Nakanishi, Kin-ya Tomizaki, Tomohiro Umetani, Fumihiro Kayamori

**Affiliations:** 1 Faculty of Frontiers of Innovative Research in Science and Technology (FIRST), Konan University, Kobe, Japan; 2 Research Institute for Nanobio-Environment and Non-Ionizing Radiation (RINNIR), Konan University, Kobe, Japan; 3 Beyond5G, Donated Lectures, Konan University, Kobe, Japan; 4 Minato Medical Science Co. Ltd., Osaka, Japan; 5 Seikoh Giken Co. Ltd., Matsudo, Japan; 6 DSP Research, Inc., Kobe, Japan; 7 Department of Materials Chemistry, Ryukoku University, Otsu, Japan; 8 Innovative Materials and Processing Research Center, Ryukoku University, Otsu, Japan; 9 Faculty of Intelligence and Informatics, Konan University, Kobe, Japan

**Keywords:** Environmental chemistry, Risk factors, Peptides, Calcium, Chemical engineering, Biomaterials, Microwave chemistry, Process chemistry, Chemical engineering, Biomaterials, Nanoscale materials, Nanobiotechnology, Nanoscale materials

## Abstract

Microwaves are used for diverse applications such as mobile phones, ovens, and therapy devices. However, there are few reports on the effects of microwaves on diseases other than cancer, and on physiological processes. Here, we focused on CaCO_3_ mineralization as a model of biomineralization and attempted to elucidate the effect of microwaves on CaCO_3_ mineralization using peptides. We conducted AFM, ζ potential, HPLC, ICP-AES, and relative permittivity measurements. Our findings show that microwaves alter the nanomorphology of the CaCO_3_ precipitate, from sphere-like particles to string-like structures. Furthermore, microwaves have little effect on the mineralization when the mineralization ability of a peptide is high, but a large effect when the precipitation ability is low. Our findings may be applicable to not only the treatment of teeth and bones but also the development of organic–inorganic nanobiomaterials. This methodology can be expanded to other molecular/atomic reactions under various microwave conditions to alter reaction activity parameters.

## Introduction

Microwaves (MWs) are used for diverse applications such as mobile phones, MW ovens, and MW therapy devices. However, MWs generated by these devices are subject to international regulations due to concern regarding their effects on the human body. For example, there are several reports that people exposed to MWs for a long period have a high prevalence of cancer^1^, and those who extensively use mobile phones have a high prevalence of brain tumors^2^. On the other hand, a report summarizing 219 epidemiological research papers on the effects of brain tumors in young people did not show an increase in morbidity due to MW exposure resulting from the use of mobile phones^3^. MWs have high bio-permeability and heating capacity, and thus are used for orthopedic warming products and liver cancer treatment^4^. MWs are also used in the synthesis of organic materials^5–8^, inorganic materials^9–12^, and peptides^13,14^. However, in the life science field, there are few research reports on the effects of MWs on diseases other than cancer, or on physiology. Diseases and physiology affect the responses of cells due to complex interactions of biomolecules such as proteins and peptides. Therefore, the behavior of various molecules under MW irradiation requires detailed analysis to elucidate the effects of MWs on diseases and biological functions. In this study, we focused on calcium carbonate (CaCO_3_) mineralization as a model biological reaction^15–23^, the process by which the exoskeletons of crustaceans, teeth, and bone are formed. Biomineralization is the precipitation of inorganics by biomolecules such as proteins and peptides^24^. We previously focused on CaCO_3_ precipitation using peptides and attempted to elucidate aspects of the mechanism underlying biomineralization by modifying the *N*-terminus in the core sequence of calcium carbonate (CaCO_3_) precipitating peptides (CAP-1 sequence, a part of the crayfish exoskeleton)^15^. Investigating the effect of MWs on CaCO_3_ biomineralization using peptides would help elucidate the behavior of both organic and inorganic molecules, providing clues on the effect of MWs on biological processes. We prepared a semiconductor transmitter (Minato Medical Science Co., Ltd.) to generate linearly polarized (directional) MWs and analyzed the correlation between the MW output watt and morphology, ζ potential, precipitation, peptide consumption, etc., on mineralization using peptides. These experiments will provide a more detailed understanding of the relationship between MW parameters and biomineralization parameters, which will be applicable to not only the treatment of teeth and bones but also the development of inorganic nanomaterials. In addition, this study provides insights into the effects of MWs on other molecules, and these effects could be controlled by changing MW parameters such as irradiation polarization and output watts.

## Results

### Design of CaCO_3_ precipitating peptides in this study

**Figure 1:**
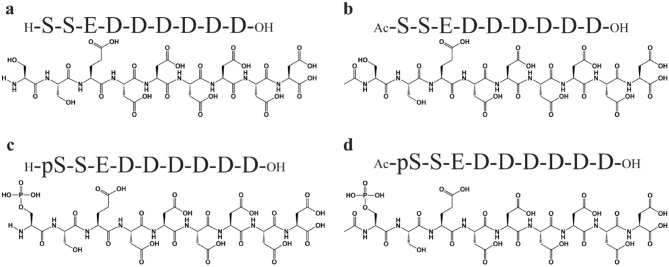
Peptide sequences and structures. Sequences of the CaCO_3_ precipitating peptides, (**a**) S peptide, (**b**) Ac-S peptide, (**c**) pS peptide, and (**d**) Ac-pS peptide, used in this study.

First, we selected the peptides for this study. We previously analyzed the effects of CaCO_3_ mineralization in detail using four peptides with different net negative charges by modifying the *N*-terminus in the core sequence of CaCO_3_ precipitating peptides (CAP-1 sequence, a part of the crayfish exoskeleton)^15,23^ (Fig. 1). Briefly, we modified the core sequences by *N*-terminus phosphorylation and/or *N*-terminus acetylation or left the *N*-terminus unmodified. We estimated the change in the CaCO_3_ precipitation ability of each peptide using inductively coupled plasma-atomic emission spectroscopy (ICP-AES). These peptides showed different abilities for CaCO_3_ precipitation (Fig. S1). The order of higher precipitation ability was Ac-S peptide ≈ > Ac-pS peptide > pS peptide > S peptide.

### Manufacture of MW irradiation equipment

**Figure 2:**
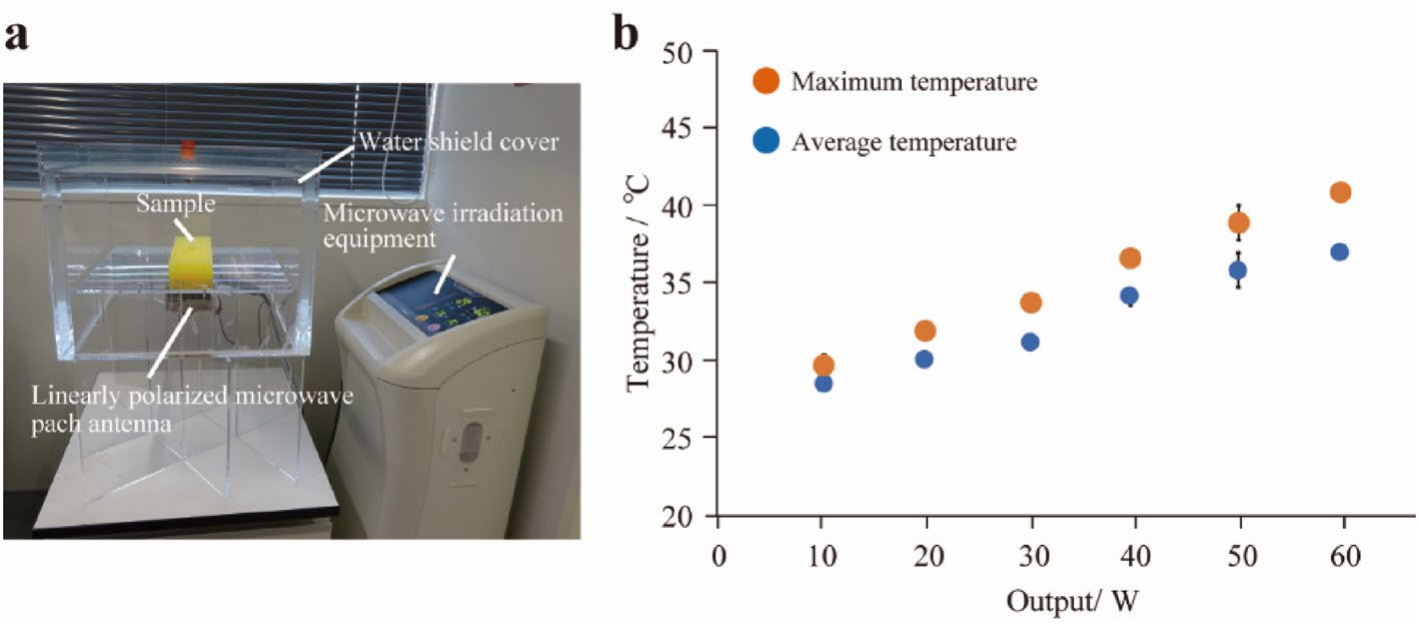
MW irradiation equipment. (**a**) MW irradiation equipment used in this study. (**b**) Solution temperature variation upon changing output watt of the MW irradiation equipment.

A semiconductor-type transmitter was used as the MW source, and a linearly polarized antenna was placed to irradiate MWs from below the sample (Fig. 2a). General MW devices such as MW ovens and medical MW therapy devices generate MWs using a magnetron. MWs oscillated by the magnetron have a wide frequency band. Moreover, the center frequency of oscillation can fluctuate when the output is analog-controlled. Here, we manufactured a semiconductor MW generator consisting of a digital frequency-controlled oscillator and a semiconductor amplifier to minimize the effects of fluctuation factors such as frequency. In addition, the output energy is controlled by a duty controller by taking advantage of the characteristics of the semiconductor. This MW irradiation device thus outputs a narrower band without frequency fluctuation than those of magnetron devices.

We then analyzed E-field strength of the MW irradiation equipment and checked polarization direction at the MW irradiated sample. However, the E-field strength distribution generated by this small MW irradiation device is not uniform over a wide range. Consequently, the E-field measuring device requires spatial resolution of the E-field strength, frequency, and polarization direction in and around the micro-tube containing the test sample solution, and the spatial resolution should be less than or equal to the size of the micro-tube. Therefore, as an E-field measurement device, we used an optical E-field sensor with an optical waveguide formed on a small Pockels element and a tiny dipole antenna formed by opposing triangular metal thin films with a base of 1.5 mm × height of 1.2 mm. The optical characteristics of the element are altered by the E-field received by the antenna (electro-optical effect). The E-field generated by passing a laser beam through the optical waveguide directly optically modulates the laser beam, which then passes through the optical fiber. This allows measurement of the E-field strength, frequency, and polarization direction without being disturbed by the MWs irradiating the measurement signal. The polarization direction was vertical to a major axis of the antenna of the MW irradiation system. Additionally, this MW irradiation device output a significantly narrow band with no frequency fluctuation (Fig. S2a). These results suggested that this MW irradiation equipment can generate an E-field strength with an exact 2.45 GHz frequency and linear polarization, allowing detailed analysis of the relationship between MWs and the behavior of biomolecules involved in biomineralization.

Next, we measured the temperature of the sample solution under MW irradiation using a thermocouple thermometer because MWs locally heat the sample. The average temperature and the maximum temperature increased gradually with the output of watts of the MW irradiation equipment (Figs. 2b, S2b). The average temperature at the sample solution after MW irradiation at a maximum output watt (60 W) was 37.2 °C and the maximum temperature was 41.1 °C.

### Change in morphology of mineralized CaCO_3_ with MW irradiation

**Figure 3:**
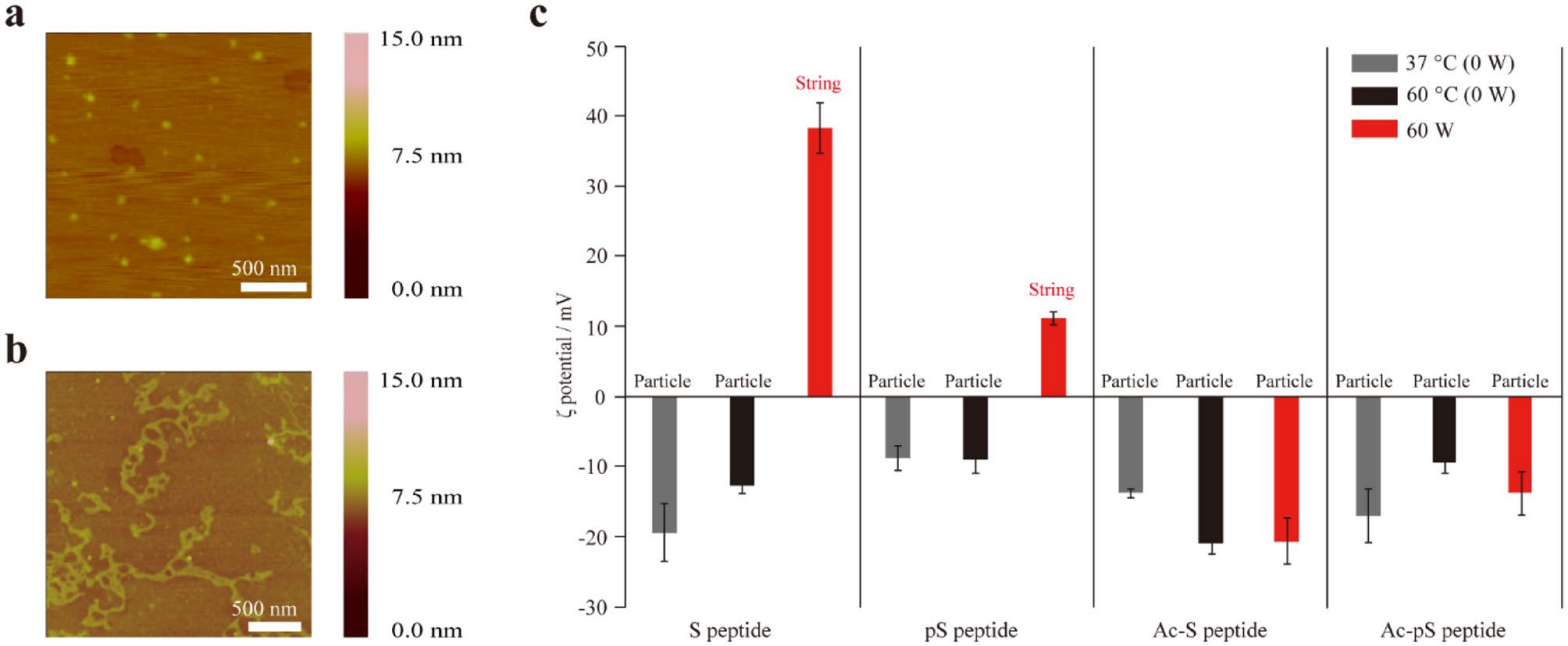
Change in morphology with MW irradiation. (**a**) AFM image of CaCO_3_ precipitates using 100 μM S peptide at 37 °C under non-MW irradiation (representative of particle image). (**b**) AFM image of CaCO_3_ precipitates using 100 μM S peptide under MW irradiation at 60 W (representative of string image). (**c**) ζ potentials of the samples after CaCO_3_ mineralization using 10 μM peptides under MW irradiation and non-irradiation conditions.

The CaCO_3_ precipitates formed under MW irradiation and non-irradiation conditions at a peptide concentration of 100 µM were observed by atomic force microscopy (AFM) and transmission electron microscopy (TEM). AFM images showed that CaCO_3_ mineralized using S peptide mostly form sphere-like precipitates in the absence of irradiation (MW non-irradiation) at 37 °C (Figs. 3a, S3e) and string-like precipitates under MW irradiation (Figs. 3b, S3a). We also checked CaCO_3_ scarcely seem to be precipitated without peptides (Fig. S3i). TEM images showed the formation of string-like precipitates under MW irradiation (Fig. S3j) and the formation of particulate precipitates under MW non-irradiation conditions at 37 °C, 60 °C, and 90 °C (Fig. S3k–m), which almost corresponded to the AFM images. These results suggest that CaCO_3_ precipitation occurs under MW irradiation by a precipitation mechanism different from that under MW non-irradiation conditions. Experiments were conducted with peptide sequences known to have higher precipitation ability than S peptide (Fig. S1). In contrast, the results hardly showed formation of string-like CaCO_3_ precipitates under MW irradiation with pS peptide, Ac-S peptide, and Ac-pS peptide (Fig. S3b–d), or under MW non-irradiation conditions at 37 °C (Fig. S3f–h). We therefore gradually decreased the concentration of each peptide and observed the morphology of the CaCO_3_ precipitates under MW irradiation. At 10 µM peptide concentration, both S peptide and pS peptide resulted in string-like precipitates under MW irradiation conditions (Fig. S4a,b), whereas sphere-like precipitates were dominantly observed using the other peptides under MW irradiation (Fig. S4c,d). All the peptide conditions hardly provided string-like precipitates under MW non-irradiation (Fig. S4e–h). S peptide at 1 µM did not cause CaCO_3_ precipitation due to its low precipitation ability, but string-like precipitates could be seen with all other peptides under MW irradiation conditions (Fig. S5a–d). Particle precipitates were mostly observed at room temperature or with heating without MW irradiation (Fig. S5e–h). These results suggest that string-like precipitates may form even at 100 μM for peptides with low precipitation ability, whereas string-like precipitates may form only at 1 µM for peptides with high precipitation ability (Table 1).

**Table 1:** Relationship between precipitation morphology and MW irradiation at 60 W using various peptides at different concentrations.

Peptide conc	S peptide		pS peptide		Ac-S peptide		Ac-pS peptide	
	Heating	MW	Heating	MW	Heating	MW	Heating	MW
100 µM	Particle	String	Particle	Particle	Particle	Particle	Particle	Particle
10 µM	Particle	String	Particle	String	Particle	Particle	Particle	Particle
1 µM	–	–	Particle	String	Particle	String	Particle	String

Next, the surface potential of CaCO_3_ formed under MW irradiation and non-irradiation conditions using 10 µM peptide was determined by ζ potential measurements. The ζ potential values were negative for all peptides under MW non-irradiation conditions (Fig. 3c). In contrast, under MW irradiation conditions, the ζ potential values were positive under conditions resulting in string-like precipitates (S peptide and pS peptide), and the ζ potential values were negative under conditions resulting in sphere-like precipitates (Ac-S peptide and Ac-pS peptide) (Fig. 3c). These results suggested that peptides were attached to the surface of the sphere-like precipitates and calcium was attached to the surface of the string-like precipitates since these peptides were negatively charged and calcium ions (Ca^2+^) were positively charged.

**Figure 4:**
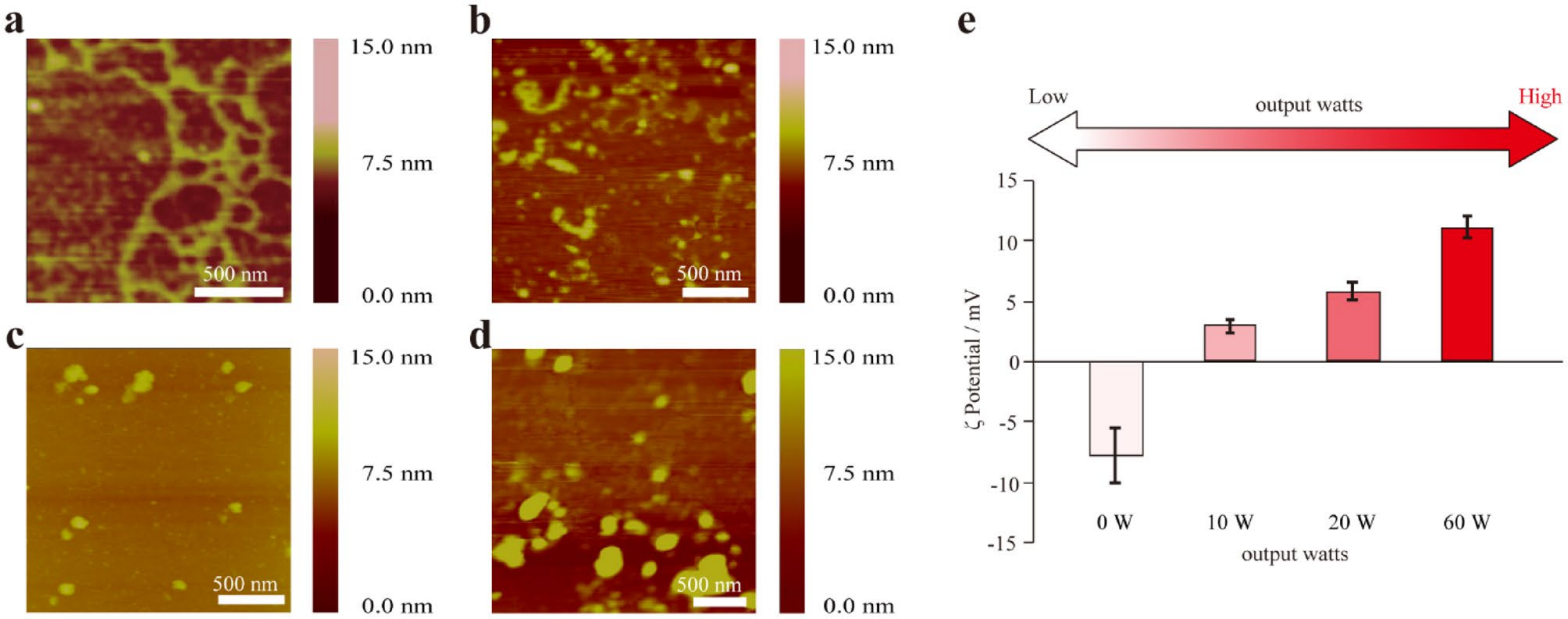
Change in morphology with the output watts. (**a**) 10 µM pS peptide (60 W). (**b**) 10 µM pS peptide (20 W). (**c**) 10 µM pS peptide (10 W). (**d**) 10 µM pS peptide (0 W). (**e**) ζ potential of the samples after CaCO_3_ mineralization under various output watts.

We then gradually decreased the output watts (60, 20, 10, and 0 W) used for MW irradiation and confirmed the morphologies and ζ potentials of the CaCO_3_ precipitates using 10 µM pS peptide. The AFM image at 60 W showed only string-like precipitates (Figs. 4a, S4b, S6g), whereas that at 20 W showed a mixture of string-like precipitates and spherical particles (Figs. 4b, S6h). The AFM images at both 0 and 10 W showed spherical particles (Figs. 4c,d, S3f, S6i,j). The ζ potential values decreased gradually from positive to negative as the output watts decreased, and the samples with more particle-like precipitates showed more negative ζ potentials. This was consistent with the results above derived from the 10 µM peptides (Figs. 3c, S4).

In the case of peptides whose precipitation abilities were relatively high, 1 µM was selected as the peptide concentration for the ζ potential analysis (decreasing the output watts (60, 20, 10, and 0 W) used for MW irradiation). ζ potential values decreased gradually from positive to negative as the output watts decreased (Fig. S6a,b), similarly to the 10 µM pS peptide analysis (Fig. 4e). In case of peptides whose precipitation abilities were relatively low, 100 µM was selected as the peptide concentration for the ζ potential analysis (decreasing the output watts (60, 20, 10, and 0 W) used for MW irradiation). The ζ potential values decreased gradually from positive to negative as the output watts decreased similar to the other peptide analysis (Fig. S6c). When MW power was decreased, we also confirmed the morphology changed from string to particle for 1 µM Ac-S peptide, 1 μM Ac-pS peptide, and 100 μM S peptide by AFM (Fig. S6d–f). These results implied that peptides with lower mineralization ability and/or a lower concentration of peptide influenced the result of MW irradiation (string-like morphology and positive ζ potential), and that a stronger MW output influenced mineralization.

### Effects of MW on Ca^2+^ and the peptides under mineralization

**Figure 5:**
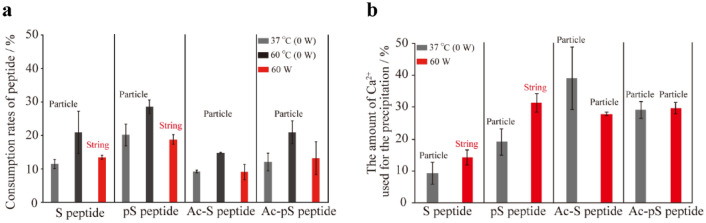
Effects of MW on Ca^2+^ and the peptides under mineralization. (**a**) Consumption of peptide determined from the peak areas following HPLC analyses. (**b**) The amount of Ca^2+^ used for the precipitation in various peptide samples as determined by ICP-AES.

We attempted to estimate the consumption rates of the peptides (the rate of amount of each peptide bound to or entrapped in the CaCO_3_ deposits to the initial amount of peptide) using high-performance liquid chromatography (HPLC) to determine the amount of residual peptide following precipitation (Fig. 5a). The consumption rates of all the peptides were unchanged under MW irradiation and MW non-irradiation conditions at 37 °C, whereas the consumption rates of all the peptides under MW non-irradiation conditions at 60 °C increased relative to those under MW non-irradiation conditions at 37 °C. These results suggest that MWs have lower effect on peptides.

Subsequently, we estimated the change in the CaCO_3_ precipitation ability of each peptide under MW conditions using ICP-AES. We first confirmed that the amount of Ca^2+^ used for the precipitation (amount of precipitates in the sample converted to Ca^2+^ mol) determined by ICP-AES was roughly correlated with our previous analysis by standard titration with ethylenediaminetetraacetate^23^ under non-MW irradiation conditions (Fig. 5b, gray bars). MW irradiation increased the amount of Ca^2+^ used for the precipitation under conditions (S peptide and pS peptide) resulting in the formation of string-like precipitates (Fig. 5b, red bars). In contrast, the amount of Ca^2+^ used for the precipitation was not changed under conditions (Ac-S peptide and Ac-pS peptide) resulting in the formation of sphere-like precipitates (Fig. 5b, red bars). Furthermore, we measured the relative permittivity of the Ca(HCO_3_)_2_ solution and each peptide to determine their susceptibility to MW irradiation. All peptides showed similarly lower relative permittivity values, whereas the relative permittivity of Ca(HCO_3_)_2_ solution was at least 4 times higher than that of the peptides (Fig. S7).

All the results of HPLC, ICP-AES and relative permittivity measurements were implied that morphological changes might attribute to effect of MWs mainly to Ca^2+^ and that differences in effects of MWs on peptides was relatively low. On the other hand, CaCO_3_ did not precipitate without peptides. Thus, peptides with lower mineralization ability and/or a lower concentration of peptide influenced the morphological results of MW irradiation, which corresponded to the ζ potential analysis described above.

## Conclusions

In conclusion, significant MW effects alter the morphology of CaCO_3_ precipitates, from sphere-like particles to string-like structures. Furthermore, MWs have little effect on the mineralization of CaCO_3_ when the CaCO_3_ precipitation ability of a peptide is high, whereas MWs have a large effect when the peptide precipitation ability is low. This study sheds light on the relationship between several MW parameters, such as the output watts, and several mineralization peptide parameters, such as the amount of precipitation. In future studies, we will use computational chemistry to investigate the direct relationship among MWs, peptide sequences and mineralization^25,26^ and compare them to wet experimental results. Our findings may aid in the treatment of teeth and bones and the development of organic–inorganic nanobiomaterials. Furthermore, the findings described herein are not limited to nano-mineralization but can be expanded to other molecular/atomic reactions under various MW conditions such as the output watts to alter reaction activity parameters.

## Materials and methods

### General remarks

All chemicals and solvents were of reagent or HPLC grade and were used without further purification. HPLC was performed on a GL-7400 HPLC system (GL Sciences, Tokyo, Japan) using an Inertsil ODS-3 column (10 × 250 mm; GL Sciences) for preparative purification, with a linear acetonitrile/0.1% trifluoroacetic acid (TFA) gradient at a flow rate of 3.0 mL/min. Peptides were analyzed using MALDI-TOF MS on an Autoflex III (Bruker Daltonics, Billerica, MA, USA) mass spectrometer with 3,5-dimethoxy-4-hydroxycinnamic acid as the matrix. Amino acid analysis was conducted using an Inertsil ODS-2 column (4.6 × 200 mm; GL Sciences) after samples were hydrolyzed in 6 M HCl at 110 °C for 24 h in a sealed tube and then labeled with phenylisothiocyanate.

### Peptide synthesis

The peptides were synthesized manually on Wang resin using the DIPCI (*N,N'*-diisopropylcarbodiimide)—DMAP (*N,N*-dimethyl-4-aminopyridine) method for the first residue, and Fmoc solid-phase peptide synthesis^27^ with 2-(1*H*-benzotriazole-1-yl)-1,1,3,3-tetramethyluronium hexafluorophosphate (HBTU, Watanabe Chemical, Hiroshima, Japan)—1-hydroxybenzotriazole (HOBt, Watanabe Chemical) for the subsequent residues. Fmoc deprotection was conducted using 1 % HOBt and 25 % piperidine in NMP (*N*-methylpyrrolidone) Side-chain protection was *O*-*t*-butyl (﻿*Ot*Bu) for Asp, Glu, and Ser, and *O*-benzyl (*O*Bzl) for phosphoserine (pSer). Peptides were cleaved from the resins and side-chain protection was removed by incubating the peptide-resin for 2 h in TFA (Watanabe Chemical Industries)/H_2_O/triisopropylsilane (Wako Pure Chemical Industries, Osaka, Japan) (20:1:1, v/v). The peptides were precipitated by the addition of cold diethyl ether and collected by centrifugation. The peptides were purified by RP-HPLC and characterized by amino acid analysis and MALDI-TOF MS: S peptide, *m/z* 1012.8 ([M + H]^+^ calcd. 1012.8); pS peptide, *m/z* 1092.8 ([M + H]^+^ calcd. 1093.3); Ac-S peptide, *m/z* 1052.4 ([M − H]^−^ calcd. 1052.8); Ac-pS peptide, *m/z* 1132.3 ([M − H]^−^ calcd. 1132.8). The peptides were dissolved in MilliQ water to about 1 mM, and the concentration was determined by amino acid analysis. The peptide solutions were stored at 4 °C.

### MW irradiation system

Schematics and a photograph of the microwave device used in this study are shown in Fig. 2a. A custom semiconductor-type microwave (MW) generator system was manufactured by Minato Medical Science Co., Ltd., Osaka, Japan). The minimum–maximum output power used in this study was 10–60 W (10 W steps); power was output to an AC transformer type system; the oscillation frequency was 2450 ± 5 MHz [a compensation value, measured value was 2450 ± 3 MHz (Fig. 2a)]; the antenna was a patch antenna (linearly polarized wave) VSWR < 1.4; 9–45% (10–50 W) for duty ratio. Note that the semiconductor generator was connected using a coaxial waveguide converter, and the MW power loss that occurred was corrected in advance. MW irradiation was performed as follows: (1) MW irradiation for 80 min; (2) Removing the water shield cover and leaving at room temperature for 20 min; (3) MW irradiation for 80 min.

### Electric field strength measurements

The E-fields of the irradiated MWs from the generators were monitored using a sensor head (6 × 6 × 23 mm, ES-100, Seikoh Giken Co., Ltd., Matsudo, Japan) and a controller (C5-D1-A, Seikoh Giken Co., Ltd.). The E-field strength [dBμV/m] was obtained using the following calculation:

$${\text{E }}\left[ {{\text{dB}}\upmu {\text{V}}/{\text{m}}} \right] = {\text{P }}\left[ {{\text{dBm}}} \right] + {\text{AF }}\left[ {\text{dB/m}} \right] + 107 \, ({\text{P}}:{\text{ Controller }}\;{\text{output }}\left[ {{\text{dBm}}} \right];{\text{ AF}}:{\text{ Antenna }}\;{\text{factor }}\left[ {\text{dB/m}} \right]).$$

### Temperature measurements

A thermocouple thermometer (Card Logger MR5300, MR9302, CHINO Corp., Tokyo, Japan) was inserted into a micro-tube containing 1 mL of MilliQ water and the temperature change due to MW irradiation at 10–60 W was measured. Previously, we had checked the temperature had been almost the same as the thermocouple measurement by the time-point measurement using thermography (OPTXI40LTF20CFT090, Optris GmbH, Berlin, Germany).

### CaCO_3_ mineralization under MW irradiation and non-irradiation

Prior to CaCO_3_ precipitation, CaCO_3_ (0.5 mmol) was suspended in MilliQ water (30 mL). CO_2_ gas was bubbled into the stirred suspension for 1 h, then the remaining solid CaCO_3_ was removed by filtration. The concentration of Ca^2+^ in the solution was determined by standard titration with ethylenediaminetetraacetate^22,23^. CaCO_3_ precipitation was conducted in a micro-tube. The Ca(HCO_3_)_2_ solution and peptide solution were diluted and mixed to the desired concentration with MilliQ water and incubated for 3 h. MW irradiation was performed as follows: (1) MW irradiation for 80 min; (2) Remove the water shield cover, leave at room temperature for 20 min; (3) MW irradiation for 80 min. Under MW non-irradiation condition, the Program Temp Control System (PC708, ASTEC, Fukuoka, Japan) was used for heating at 37 °C, 60 °C, and 90 °C.

### AFM measurements

The entire volume of each sample was placed on freshly cleaved mica (1 × 1 cm). After 5 min, the solvent was absorbed with filter paper. MilliQ water (20 µL) was then placed on the mica surface and immediately absorbed with filter paper. This process was repeated three times to remove salts from the sample. All samples were dried in vacuo prior to AFM measurements. Tapping-mode images were obtained on a multimode scanning probe microscope with a Nanoscope IIIa controller (Veeco, Woodbury, NY, USA).

### TEM measurements

The CaCO_3_ precipitation sample (20 µL) was placed on a TEM grid for 1 min and dried with filter paper. MilliQ water (20 µL) was then placed on the grid and immediately absorbed with filter paper. This process was repeated three times. All samples were dried in vacuo prior to TEM measurements, which were conducted at an accelerating voltage of 115 kV (JEM-1400, JEOL, Tokyo, Japan).

### ζ potential measurements

Sample solution (750 mL for ζ potential) was transferred into a folded capillary cell (DTS1070, Malvern Instruments, Worcestershire, UK) for ζ potential measurements. ζ potential data were acquired on a Zetasizer ZEN3600 instrument (Sysmex, Kobe, Japan) equipped with a 633 nm laser.

### HPLC analyses

Following CaCO_3_ precipitation (10 µM peptide, 150 µM Ca(HCO_3_)_2_), 300 µL MilliQ water (containing 0.1% TFA) was added to a 1.2 mL sample. The 400 µL sample was filtered with a centrifugal filter (Durapore®-PVDF 0.22 µm Ultrafree®-MC-GV, Merck, Tokyo, Japan), then RP-HPLC analysis was performed by injecting a 1 mL sample onto an Inertsil ODS-3 column (4.6 × 150 mm; GL Science) and eluting with a linear acetonitrile/0.1% TFA gradient at a flow rate of 1.0 mL/min.

### ICP-AES measurements

Following CaCO_3_ precipitation (10 µM peptide, 150 µM Ca(HCO_3_)_2_), 2 mL samples were filtered with a centrifugal filter (Durapore®-PVDF 0.22 µm Ultrafree®-MC-GV). After CaCO_3_ mineralization, samples were pyrolyzed with 5 mL of 1 mM CH_3_COOH and 5 mL of HClO_4_ for 1 h at 120 °C^28^. After pyrolysis, the samples were dissolved with 1 mL of 6 mM HCl and 9 mL of MilliQ water. Calibration curves for each element were obtained using a Ca standard solution (for atomic absorption spectrochemical analysis, Fujifilm Wako Pure Chemical Industries) in the range of 0 ppm to 10 ppm. Ca was detected at a wavelength of 389.785 nm using an ICP-AES (Spectroblue® FMX36, Hitachi High-Tech Corporation Tokyo, Japan) provided by Clean Chemical Co. Ltd.

### Relative permittivity measurements

10 µM peptide solution (1 mL) and 150 µM Ca(HCO_3_)_2_ solution (1 mL) were transferred into the relative permittivity instrument (SH2-Z, TOYO corporation, Tokyo, Japan) provided by Hyogo Prefectural Institute of Technology. Relative permittivity data at 0.2 MHz were acquired.

## Supplementary Information

Supplementary Figures.

## Data Availability

The datasets used and/or analysed during the current study available from the corresponding author on reasonable request.
