# Supplementary material for: Effect of linearly polarized microwaves on nanomorphology of calcium carbonate mineralization using peptides
Source: Sci Rep. 2023 Jul 25;13:12027. doi: 10.1038/s41598-023-37473-7 (PMC10368672; doi:10.1038/s41598-023-37473-7)
Supplement: Supplementary file 1 — Supplementary Figures. [file 41598_2023_37473_MOESM1_ESM.pdf]

Supplementary information

Effect of Linearly Polarized Microwaves on Nanomorphology of Calcium Carbonate Mineralization Using Peptides

Kenji Usui[1,2,3,10*], Makoto Ozaki[1,10], Kan Hirao[1,10], Tsubasa Kosaka[1], Natsumi Endo[1], Shuhei Yoshida[1], Shin-ichiro Yokota[1], Yonejiro Arimoto[4], Ryuji Osawa[5], Nobuhiro Nakanishi[2,3,6], Kin-ya Tomizaki[7,8], Tomohiro Umetani[2,9], Fumihiro Kayamori[1,2*]

[1] *Faculty of Frontiers of Innovative Research in Science and Technology (FIRST), Konan University, Kobe, Japan*
[2] *Research Institute for Nanobio-environment and Non-Ionizing Radiation (RINNIR), Konan University, Kobe, Japan*
[3] *Beyond5G, Donated Lectures, Konan University, Kobe, Japan*
[4] *Minato Medical Science Co. Ltd., Osaka, Japan*
[5] *Seikoh Giken Co. Ltd., Matsudo, Japan*
[6] *DSP Research, Inc., Kobe, Japan*
[7] *Department of Materials Chemistry, Ryukoku University, Otsu, Japan*
[8] *Innovative Materials and Processing Research Center, Ryukoku University, Otsu, Japan*
[9] *Faculty of Intelligence and Informatics, Konan University, Kobe, Japan*

[10] These authors contributed equally: Kenji Usui, Makoto Ozaki and Kan Hirao.

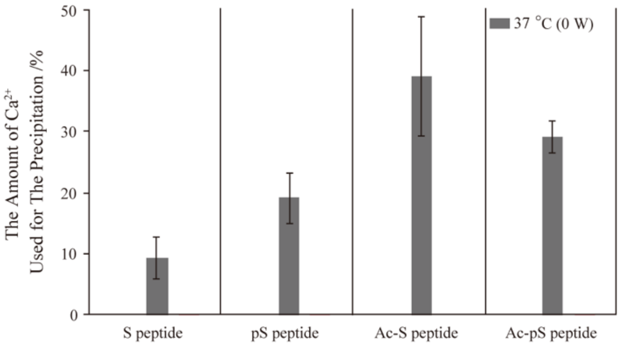

Figure S1. The amount of Ca^{2+} used for the precipitation in various peptide samples as determined by ICP-AES.

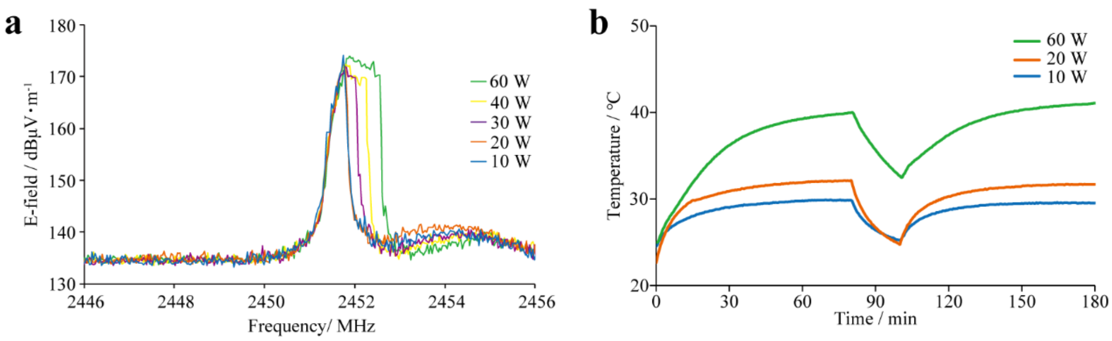

Figure S2. (**a**) Spectrum analysis of the electric field of the microwave generated from the MW irradiation device. (**b**) Representative time courses of solution temperature during the MW irradiation experiment.

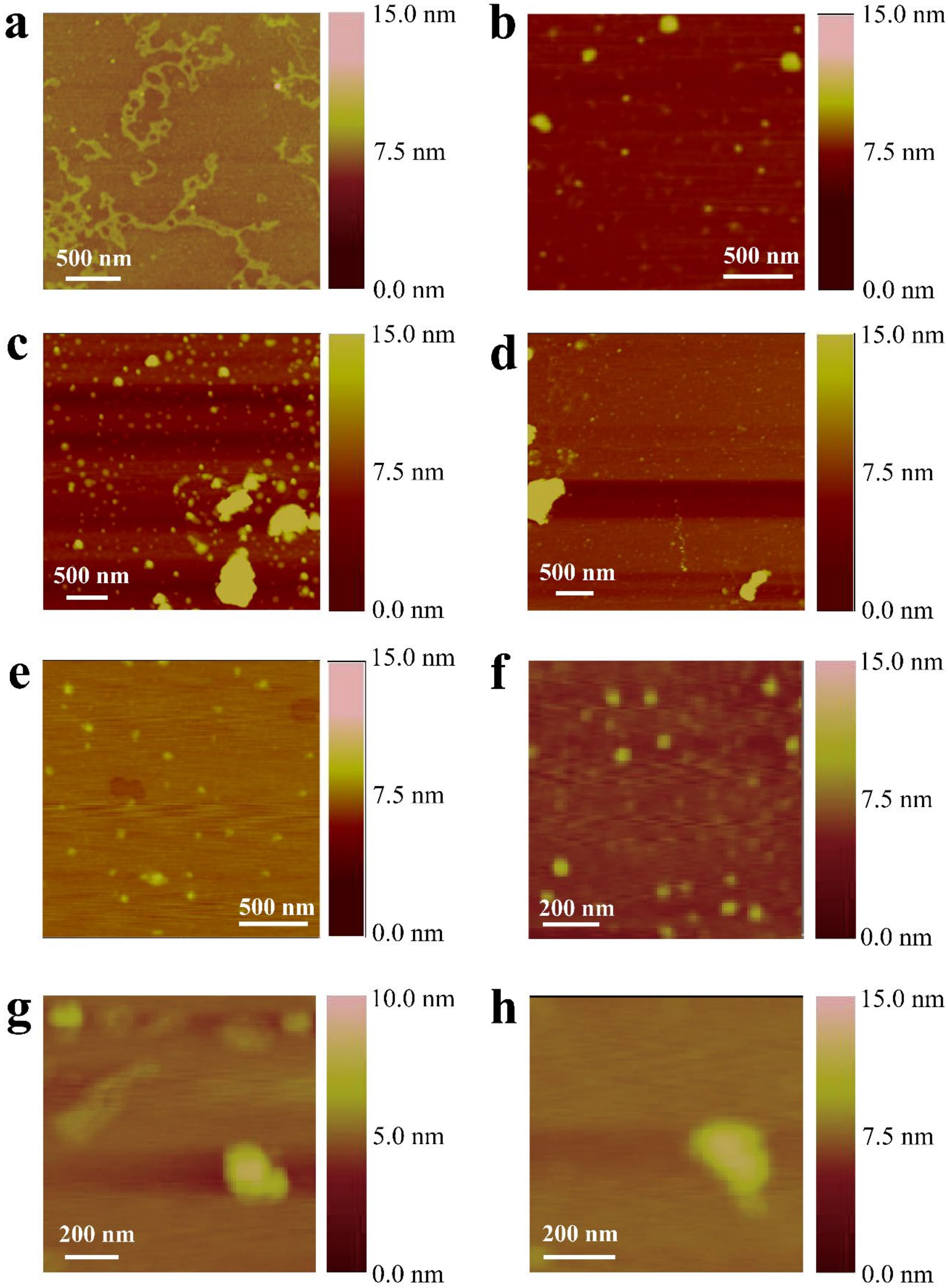
a
15.0 nm
7.5 nm
0.0 nm
500 nm
b
15.0 nm
7.5 nm
0.0 nm
500 nm
c
15.0 nm
7.5 nm
0.0 nm
500 nm
d
15.0 nm
7.5 nm
0.0 nm
500 nm
e
15.0 nm
7.5 nm
0.0 nm
500 nm
f
15.0 nm
7.5 nm
0.0 nm
200 nm
g
10.0 nm
5.0 nm
0.0 nm
200 nm
h
15.0 nm
7.5 nm
0.0 nm
200 nm

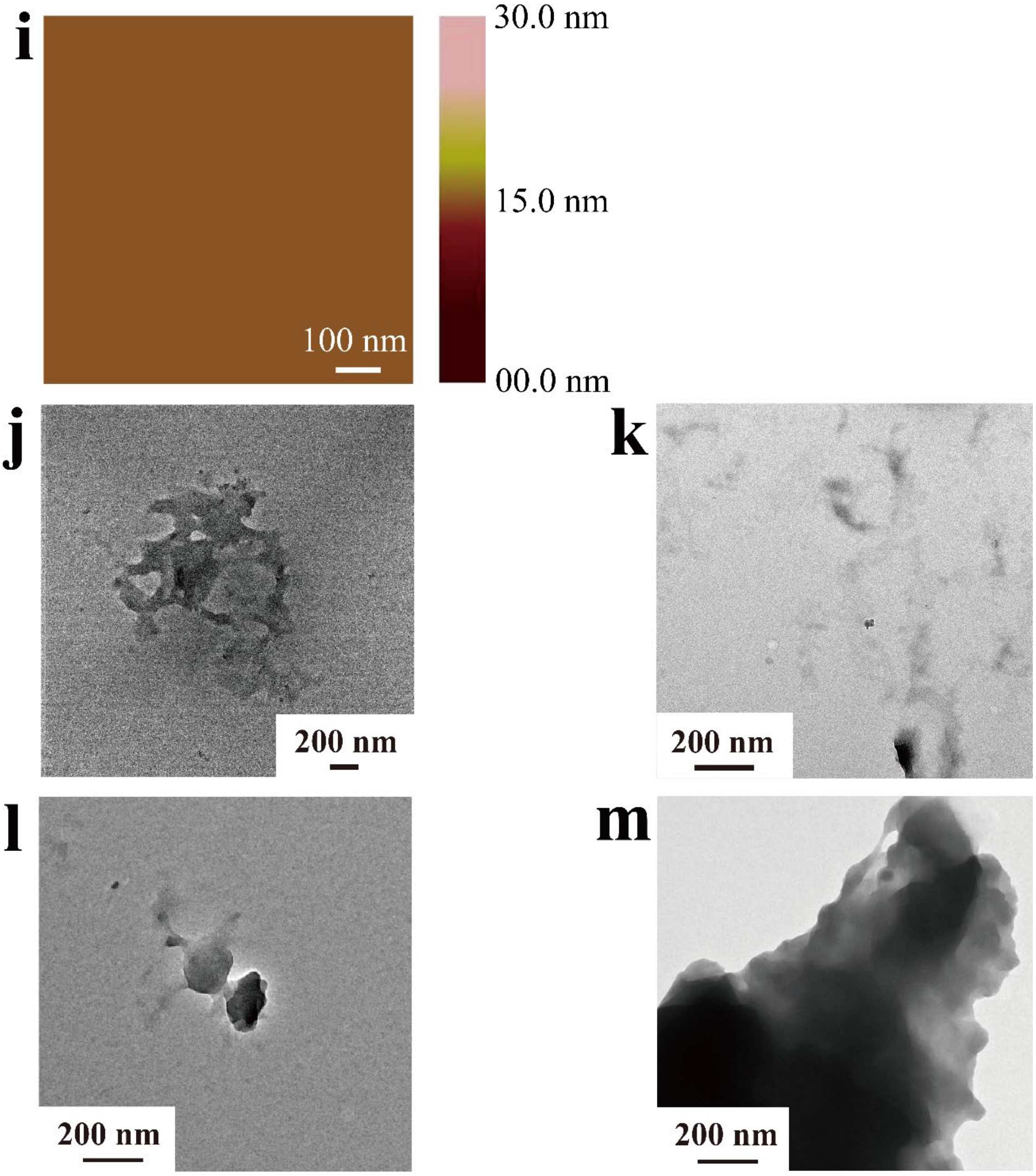

Figure S3. AFM images of the sample after $CaCO_3$ mineralization under MW irradiation at 60 W using (**a**) S peptide, (**b**) pS peptide, (**c**) Ac-S peptide, and (**d**) Ac-pS peptide. AFM images of the sample after $CaCO_3$ mineralization without MW irradiation using (**e**) S peptide, (**f**) pS peptide, (**g**) Ac-S peptide, and (**h**) Ac-pS peptide at 37 °C. (**i**) AFM image of the sample after $CaCO_3$ mineralization without peptides. (**j**) TEM image of the sample after $CaCO_3$ mineralization using S peptide under MW irradiation at 60 W. TEM images of the sample after $CaCO_3$ mineralization using S peptide at (**k**) 37 °C, (**l**) 60 °C, and (**m**) 90 °C. All samples were measured at a peptide concentration of 100 μM.

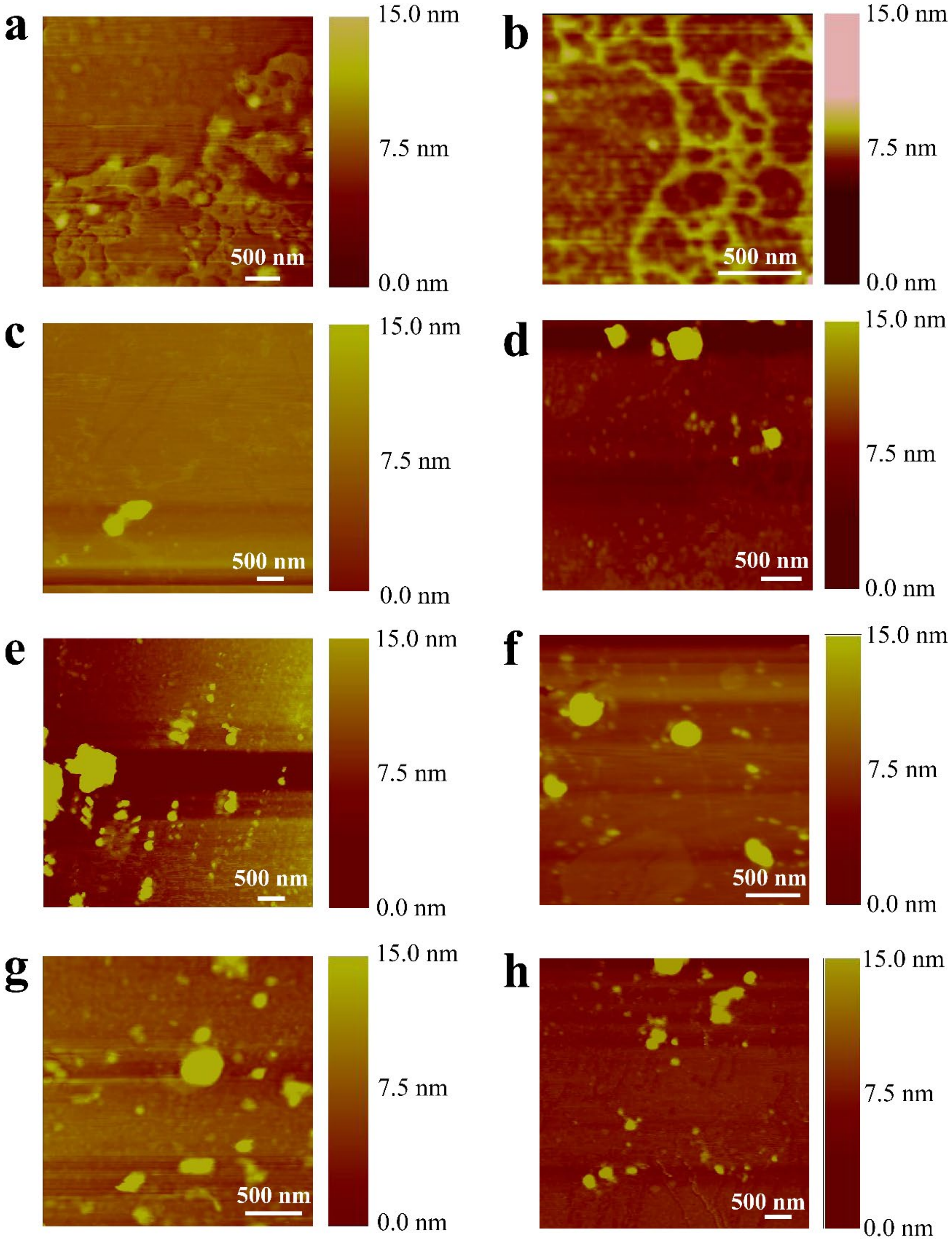

Figure S4. AFM images of the sample after $CaCO_3$ mineralization under MW irradiation at 60 W using (**a**) S peptide, (**b**) pS peptide, (**c**) Ac-S peptide, and (**d**) Ac-pS peptide. AFM images of the sample after $CaCO_3$ mineralization without MW irradiation using (**e**) S peptide, (**f**) pS peptide, (**g**) Ac-S peptide, and (**h**) Ac-pS peptide. All samples were measured at a peptide concentration of 10 µM.

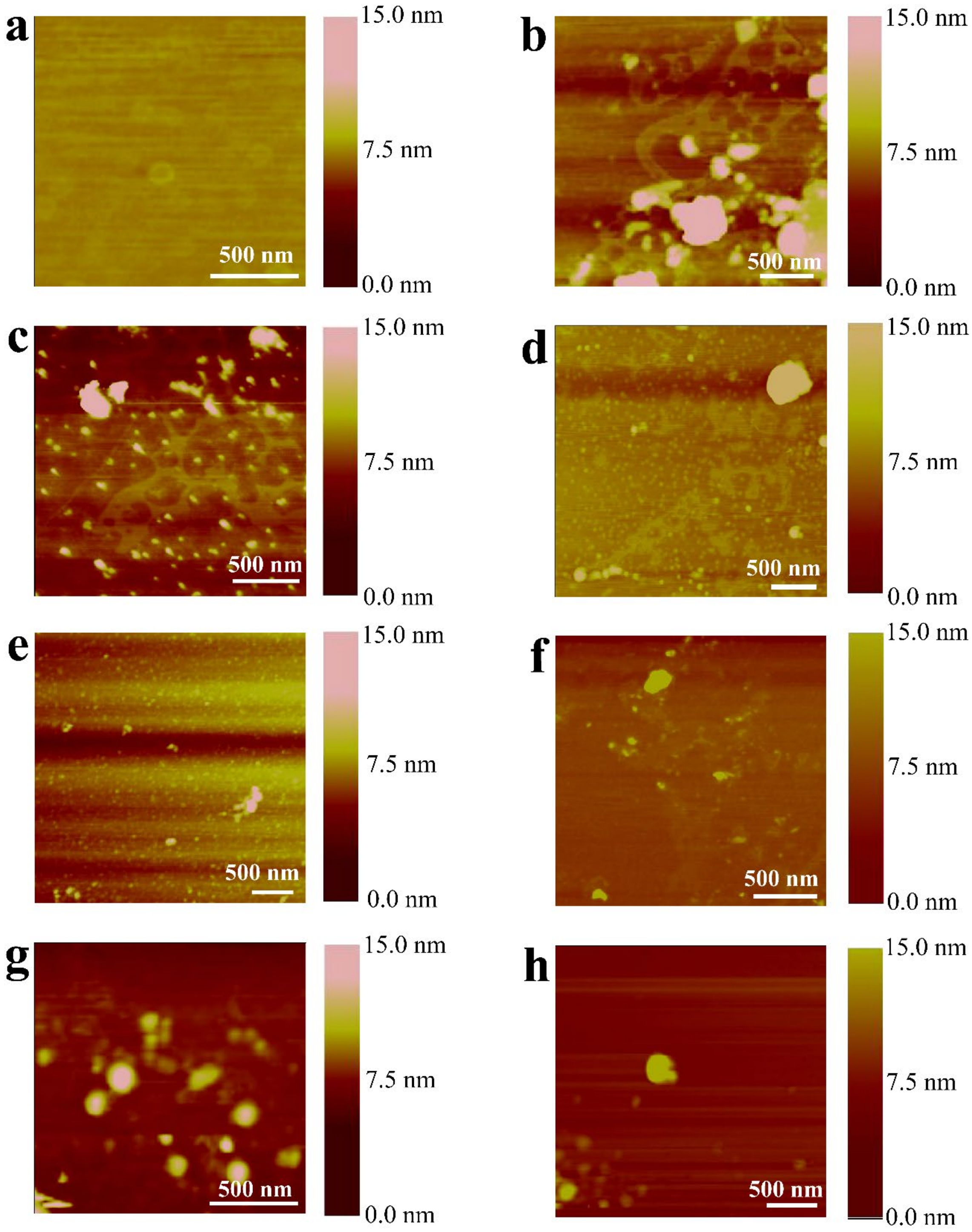

Figure S5. AFM images of the sample after $CaCO_3$ mineralization under MW irradiation at 60 W using (**a**) S peptide, (**b**) pS peptide, (**c**) Ac-S peptide, and (**d**) Ac-pS peptide. AFM images of the sample after $CaCO_3$ mineralization without MW irradiation using (**e**) S peptide, (**f**) pS peptide, (**g**) Ac-S peptide, and (**h**) Ac-pS peptide. All samples were measured at a peptide concentration of 1 μM.

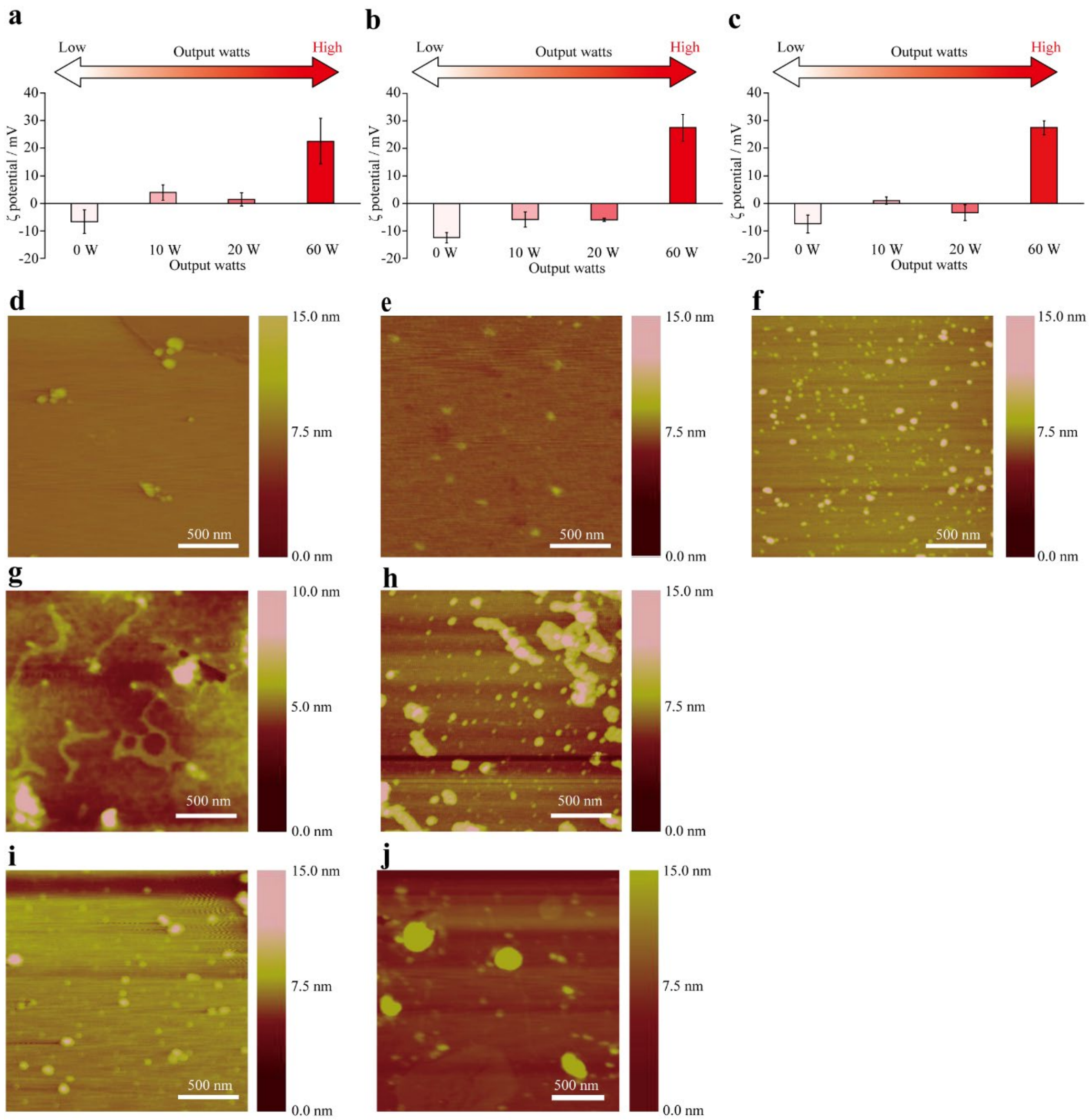

Figure S6. ζ potential of samples after $CaCO_3$ mineralization under MW irradiation with (**a**) 1 μM Ac-S peptide, (**b**) 1 μM Ac-pS peptide, and (**c**) 100 μM S peptide with the output watts changes. AFM image of the sample after $CaCO_3$ mineralization under MW irradiation at 10 W using (**d**) 1 μM Ac-S peptide, and (**e**) 1 μM Ac-pS peptide. (**f**) AFM image of 100 μM S peptide under MW irradiation at 10 W. Additional AFM images of 10 μM pS peptide under MW irradiation at (**g**) 60 W, (**h**) 20 W, and (**i**) 10 W. (**j**) AFM image of 10 μM pS peptide without MW irradiation.

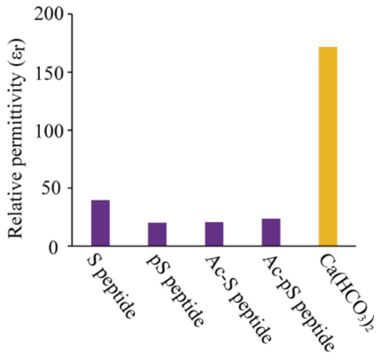

Figure S7. Relative permittivity of $Ca(HCO_3)_2$ solution and each peptide.
